# A Highly Sensitive Immunochromatographic Strip Test for Rapid and Quantitative Detection of Saikosaponin d

**DOI:** 10.3390/molecules23020338

**Published:** 2018-02-06

**Authors:** Yue Zhang, Wei Xiao, Hui Kong, Jinjun Cheng, Xin Yan, Meiling Zhang, Qingguo Wang, Huihua Qu, Yan Zhao

**Affiliations:** 1 School of Chinese Materia Medica, Beijing University of Chinese Medicine, Beijing 100029, China; 20150941177@bucm.edu.cn; 2 Jiangsu Kanion Pharmaceutical Co., Ltd., 58 Jiangning Industrial Park Kangyuan Road, Lianyungang, Jiangsu, 210000, China; kanionlunwen@163.com; 3 School of Basic Medical Sciences, Beijing University of Chinese Medicine, 11 Beisanhuandong Road, Chaoyang District, Beijing 100029, China; doris7629@126.com (H.K.); carlosjjcheng@163.com (J.C.); 20150931805@bucm.edu.cn (X.Y.); 18811790361@163.com (M.Z.); wangqg8558@sina.com (Q.W.); 4 Centre of Scientific Experiment, Beijing University of Chinese Medicine, 11 Beisanhuandong Road, Chaoyang District, Beijing 100029, China

**Keywords:** colloidal gold, immunochromatographic strip, monoclonal antibody, saikosaponin d, rapid detection

## Abstract

A quantitative lateral-flow immunoassay using gold nanoparticles (AuNPs) conjugated with a monoclonal antibody (MAb) against saikosaponin d (SSd) was developed for the analysis of SSd. The AuNPs were prepared in our laboratory. The AuNPs were polyhedral, with an average diameter of approximately 18 nm. We used the conjugation between AuNPs and MAbs against SSd to prepare immunochromatographic strips (ICSs). For the quantitative experiment, the strips with the test results were scanned using a membrane strip reader, and a detection curve (regression equation, *y* = −0.113ln(*x*) + 1.5451, *R*^2^ = 0.983), representing the averages of the scanned data, was obtained. This curve was linear from 96 ng/mL to 150 μg/mL, and the IC_50_ value was 10.39 μg/mL. In this study, we bring the concept of POCT (point-of-care testing) to the measurement of TCM compounds, and this is the first report of quantitative detection of SSd by an ICS.

## 1. Introduction

Colloidal gold nanoparticles (AuNPs), defined as gold particles with diameters ranging from 5 to 250 nm, have found widespread use in biological electron microscopy [1]. The colloidal gold immunochromatographic assay (GICA) was developed in the 1980s to detect specific compounds in various types of samples in one step without other treatments using a cellulose membrane as the carrier [2,3,4]. Based on the specific binding of the antigen and antibody, the GICA is a sensitive method for the microanalysis of antigens and antibodies [5,6]. Membrane-based lateral-flow immunochromatographic strips (ICSs) are a useful tool for low-cost and rapid detection based on the GICA [7,8,9]. The use of ICS systems does not require special training or elaborate instrumental setups. The popularity of ICSs, especially for common tests such as for pregnancy, drugs abused, and food safety, is driven by the ease of use, speed, and relative accuracy of these simple and inexpensive disposable devices [10].

Recently, ICSs have been efficiently applied to the area of bovine virus diarrhea and white spot syndrome virus detection [11,12,13,14,15,16]. Additionally, many strip tests have been applied for the detection of small molecules of, among others, streptomycin [9], chromium ions [17], paragonimiasis skrjabini [18], artesunate [8], and a number of natural products [19,20,21]. Most ICS assays can only provide qualitative (yes/no signal) or semi-quantitative results on analyte concentrations [22,23].

Radix Bupleuri (RB), a traditional Chinese medicinal formulation derived from the dried roots of *Bupleurum sinensis* DC or *Bupleurum scorzonerifolium* Willd, has been one of the most widely used herbal medicines in China, Japan, and other Asian countries for a long time [24]. According to recent research, RB has been used to counteract different inflammatory conditions, including pancreatitis, liver cirrhosis, and fever, to treat infections such as malaria and the common cold, to modulate abnormal lipid metabolism, and to relieve depression [25,26,27,28].

Saikosaponin d (SSd), authorized as a quantity control index of Radix Bupleuri along with SSa, is one of the main bioactive components in RB and possesses anti-inflammatory and immunomodulatory properties. Therefore, it is a potential chemotherapeutic drug for clinical applications [29,30]. By triggering autophagic cell death, SSd can be used to treat drug-resistant and apoptosis-resistant cancers, according to recent studies [31]. Current standard reference methods for the determination of SSd are mainly based on gas chromatography, liquid chromatography, or mass spectrometry [32,33]. In recent years, novel methods for quality surveys of SSd such as ELISA have been developed. These methods, although sensitive and accurate for detecting SSd, have shortcomings, as they require complicated pre-treatments, are time-consuming, and require sophisticated instrumentation. Thus, there is an urgent need to develop a more convenient and easy-to-use point-of-care diagnostic device for the rapid evaluation and large-scale screening of medicinal compounds.

In this article, a highly sensitive, fast-response, and low-cost competitive colloidal gold ICS was developed for the on-site detection of SSd residues. Optimization of the reaction step parameters and conditions was achieved through the modification of preparation procedures. 

## 2. Results and Discussion

### 2.1. Characterization of Colloidal Gold

**Figure 1:**
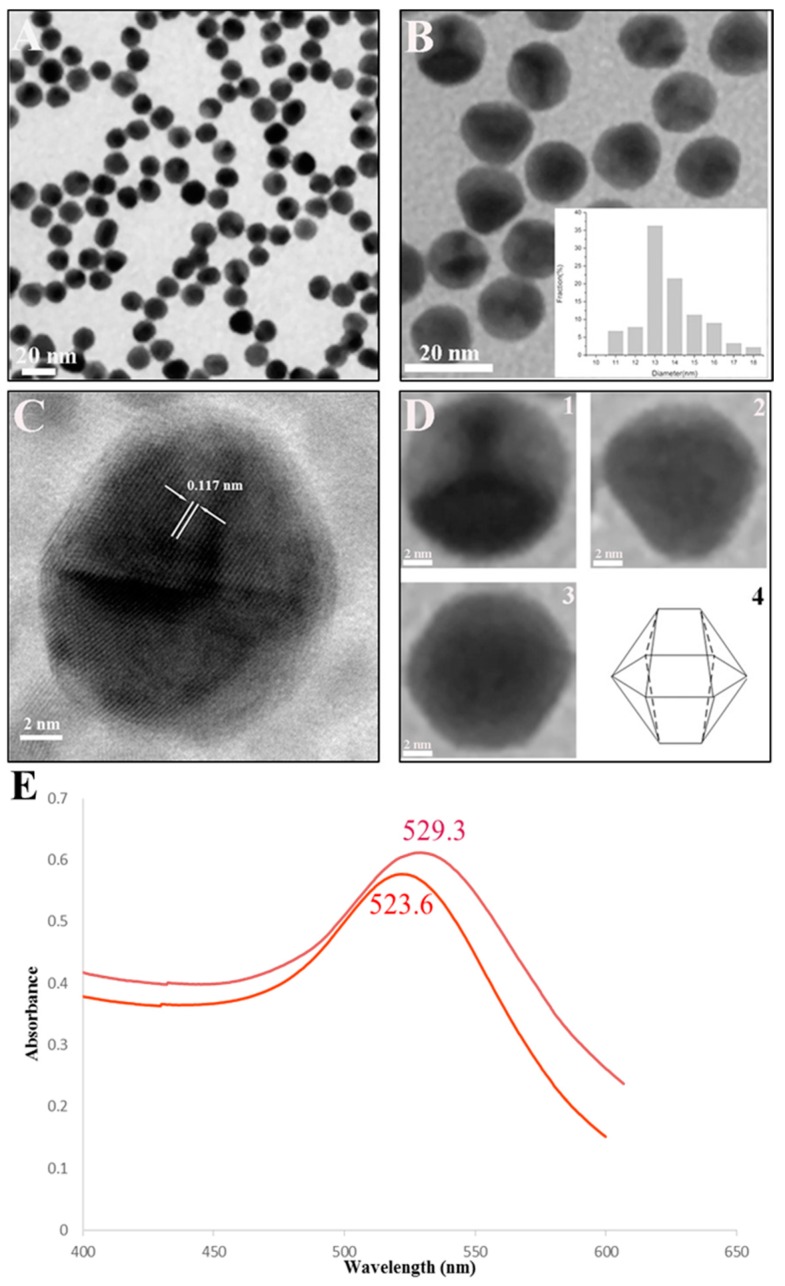
Characterization of colloidal gold. (**A**) TEM image of gold nanoparticles (AuNPs) (100,000× magnification); (**B**) TEM image of AuNPs (500,000× magnification). The size distribution of the AuNPs can be seen in the TEM image. The diameter of the AuNPs ranged from 10 nm to 20 nm, with an average of approximately 18 nm; (**C**) High-resolution TEM (HRTEM) image of AuNPs. The HRTEM image taken of an individual AuNP shows a continuous fringe pattern with a spacing of 0.117 nm; (**D**) Higher magnification images of AuNPs (1–3) and a proposed model of the AuNPs with multi-aspect optimization (4); (**E**) UV-vis absorption spectrum of pure AuNPs with a peak at 523.6 nm. (red line) and UV-vis absorption spectrum of the monoclonal antibody (MAb)–AuNP conjugates with a peak at 529.3 nm (purple line).

The prepared colloidal gold solutions were claret red. TEM analyses were used to determine the morphology and shape of the AuNPs (Figure 1A–D). Figure 1A,B revealed that the particles are polyhedral in shape and are uniformly distributed. The average diameter of AuNPs was found to be approximately 18 nm (inset of Figure 1B). A high-resolution TEM (HRTEM) image of AuNPs is shown in Figure 1C. The HRTEM image taken from an individual AuNP shows a continuous fringe pattern with a spacing of 0.117 nm. Higher magnification images of gold nanoprisms are shown in Figure 1D (1–3). A proposed model of the AuNPs with multi-aspect optimization is depicted in Figure 1D (4). The conjugation of AuNPs to anti-SSd MAbs was confirmed by spectral analysis of the pure and conjugated AuNPs in the range of 400–700 nm, in which a bathochromic shift in the peak from 523.6 to 529.3 nm was observed [34,35] (Figure 1E). 

### 2.2. Evaluation of the Strips

#### 2.2.1. Sensitivity of the ICS

**Figure 2:**
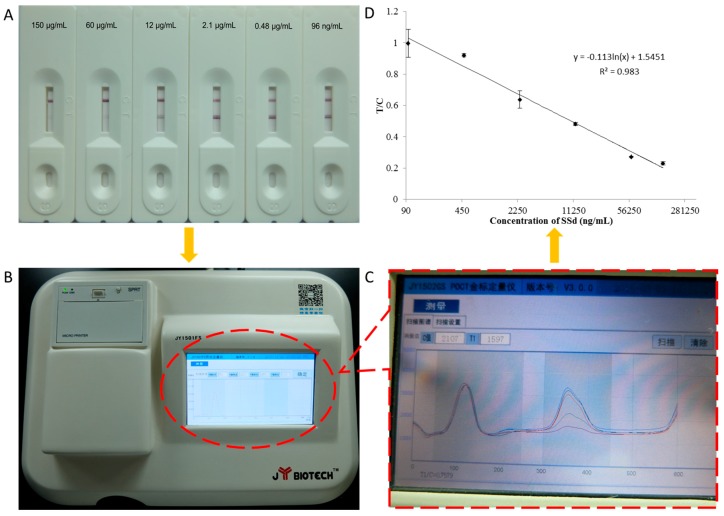
Characterization of the lateral-flow immunoassay for SSd. (**A**) Photographs of results for standard solutions containing different concentrations of SSd assayed using the ICS; (**B**) Photograph of the matched colloidal gold scanned; (**C**) The intensity pattern of the test and control lines scanned by the colloidal gold quantitative instrument; (**D**) Standard curve of icELISA for SSd determination using the ICS. The regression equation is *y* = −0.113ln(*x*) + 1.5451, with a correlation coefficient (*R*^2^) of 0.983.

Store solutions of SSd were prepared and diluted to 2 mg/mL using acetonitrile. The standard solutions were 150,000, 60,000, 12,000, 2400, 480 and 96 ng/mL of SSd in PBS, with PBS used as the control. Using the ICS, each sample was assayed at least three times. A JY1502GS portable ICS reader was used in the quantitative experiments to scan the strips with the SSd test results. The standard curve was made by relating known concentrations of SSd to the corresponding absorbance at each concentration. The linear regression equation was *y* = −0.113ln(*x*) + 1.5451, with a correlation coefficient (*R*^2^) of 0.983 (Figure 2). This curve was found to be linear from 96 ng/mL to 150 μg/mL, and the IC_50_ value was 10.39 μg/mL. The assay could be performed within 5–15 min, while the optimal test time was suggested to be 10 min in the quantitative experiment. 

#### 2.2.2. Specificity of the ICS

**Figure 3:**
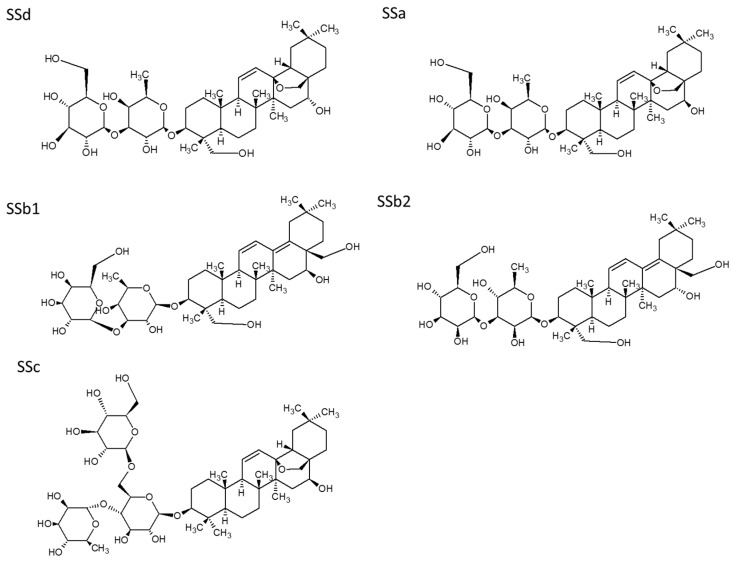
Chemical structures of saikosaponin a, saikosaponin b1, saikosaponin b2 saikosaponin c and saikosaponin d.

The specificity of the ICS was tested by evaluating its reactivity with other compounds. The ICS had low cross-reactivity with SSa and no cross-reactivity with SSc, SSb1, or SSb2 (Table 1), indicating that the ICS based on anti-SSd MAbs has high specificity. The structures of SSd and its related compounds are shown in Figure 3.

### 2.3. Recovery Rate, Repeatability, and Stability of the ICS Assay

As shown in Table 2, the average recovery rate was 113.22% (mean ± SD, *n* = 3). This method was sufficiently reliable for the determination of SSd in biological samples on the basis of its accuracy and consistency.

Assay variation was evaluated by testing the coefficients of variation (CV, %) for several concentrations of SSd by ELISA and ICS. As shown in Table S1, The RSDs were less than 4.29% in the ELISA replicates and lower than 4.48% in the ICS replicates. These results indicate that the assay variation is acceptable.

The stability of the assay was examined by means of storing the same batch of ICS assay strips for 4 and 8 weeks and then using them to detect standard SSd solutions. The results showed that the signal intensity that developed on the test line of the strip stored for 4 weeks was the same as that observed on fresh strips that were used immediately without storage (Table 3). The signals on the test and control lines of strips used for SSd detection after storage for 8 weeks had slightly weaker intensities compared to those of strips stored for 4 weeks before use. However, the coefficient of variance was lower than 6%, and the loss in intensity was determined to not be significant. Therefore, there is no question that the ICS assay remained stable after 8 weeks of storage at room temperature (25 °C).

### 2.4. Consistency between the ICS Assay and icELISA

The results obtained by the ICS assay and icELISA showed good agreement for all samples (Table 4). These results indicate that the ICS test is a useful tool for detecting SSd in traditional Chinese medicine formulations and other natural samples.

The qualitative collaurum ICS assay using anti-SSd MAbs showed high specificity for SSd. The icELISA requires a considerable amount of time to yield results. Our ICS assay could be used for the quantitative detection of SSd in 10 min. The regression curve of the scanned data and the SSd standard concentration were established in the scanning reader; therefore, a user can directly obtain the SSd concentration. The performance of the assay for sample analysis requires neither intensive labor nor expensive equipment. The assay is simple to use, and detection can be completed within 10 min; thus, the assay can be used by non-specialists. 

## 3. Materials and Methods

### 3.1. Materials

#### 3.1.1. Reagents

SSd (purity ≥ 98%) was purchased from the Shanghai Standard Biotech Co., Ltd. (Shanghai, China). SSd granules (130901H) were obtained from the Beijing Kang Ren Tang Group Co., Ltd. (Beijing, China). Bovine serum albumin (BSA) was purchased from Sigma-Aldrich (Shanghai, China). Goat anti-mouse immunoglobulin conjugated to horseradish peroxidase (GaMIgG-HRP; whole molecule) was sourced from GE Healthcare (Newark NJ, USA). Chloroauric acid solution (HAuCl_4_; purity ≥ 47.8%, based on Au) was obtained from the Tianjin Fu Chen Chemical Reagents Factory (Tianjin, China). Sodium citrate (≥99.0%) was purchased from Beijing Chemical Works (Beijing, China). Glass fiber membranes and semi-rigid PVC sheets were purchased from the Shanghai Jieyi Biological Technology Co., Ltd. (Shanghai, China). Nitrocellulose membranes were from Millipore (Boston, MA, USA), and filter paper was purchased from the Sinopharm Chemical Reagents Beijing Co., Ltd. (Beijing, China). All other chemicals were standard commercial products of analytical grade. 

#### 3.1.2. Instruments

A portable strip reader (quantitative instrument for measuring colloidal gold) was purchased from the Shanghai Jieyi Biological Technology Co., Ltd. (Model JY1502GS). The absorbance was measured on a Multiskan MK3 spectrophotometric microplate reader (Thermo Fisher Scientific, Waltham, MA, USA). Immunoreactions were carried out in a DRP-9082 electro-heating standing-temperature cultivator (Samsung Instrument Co., Ltd., Shanghai, China). A UV Probe-7200 spectrometer (Shimadzu Corporation, Tokyo, Japan) and a transmission electron microscope (JEOL, Tokyo, Japan) were used to characterize the AuNPs.

### 3.2. Methods

#### 3.2.1. Preparation of the Anti-SSd MAbs

Hybridoma cells that secreted anti-SSd antibodies had been prepared in our team in an earlier study [33]. These hybridoma cells were injected into BALB/c mice intraperitoneally to produce ascitic fluids. The titer of anti-SSd MAb was determined by ELISA, and the absorbance was measured at 450 nm by a micro-plate reader. Specificity analyses of MAbs and the purification of IgG were performed according to Sai et al. [33].

#### 3.2.2. Preparation of AuNPs

AuNPs were produced as described previously [36] with minor modifications. Briefly, 0.55 mL of 4% HAuCl_4_ and 0.132 g of sodium citrate (10 mg/mL) were quickly added to triple distilled water, which had been heated to the boiling point. After the mixture was boiled for 10 min, the color of the solution changed to wine-red. Then, the power was turned off, and the solution was cooled to room temperature (25 °C). The size and morphology of the AuNPs were determined by TEM.

#### 3.2.3. Conjugation of Anti-SSd MAbs and AuNPs

Anti-SSd MAbs and gold nanoparticles conjugate were produced as follows [37,38]. Gold colloid (1 mL) was initially adjusted to a pH of 9.0 using 0.1 M K_2_CO_3_, and 100 μL of 0.2 mg/mL anti-SSd MAbs dissolved in double-distilled water were added. The mixture was mixed for 30 min at room temperature (25 °C) under gentle stirring and then centrifuged at 2000 rpm for 10 min at room temperature (25 °C) to remove the coagulated colloidal gold. PEG 20000 (100 μL, 10% (*w*/*v*)) was added to the centrifuged solution and incubated for 10 min at 37 °C under gentle stirring, followed by another round of centrifugation at 9500 rpm for 25 min at room temperature (25 °C) to remove the excess proteins. The obtained pellet was re-suspended in 100 μL of Tris-HCL (pH = 8.8) containing 1% (*w*/*v*) BSA, 0.5% (*v*/*v*) Tween 20, and 1% (*v*/*v*) PEG 20000, and it was stored at 4 °C before use. The size and morphology of the anti-SSd MAb–AuNP conjugates were determined using TEM.

#### 3.2.4. Preparation of the Capture Reagent

SSd (5 mg) was dissolved in 1 mL of water and dropped in 1 mL of NaIO_4_ solution (5 mg/mL). The mixture was stirred at room temperature (25 °C) for 1 h. Next, 1 mg of BSA was added, and stirred for another 6 h. The mixture was dialyzed 6 times against water for 3 days, and the resultant SSd–BSA conjugate was stored at 4 °C before use. The SSd–BSA conjugate and goat anti-mouse IgG were used as the test and control capture reagents, respectively.

#### 3.2.5. Preparation of the ICS Assay 

SSd–BSA and goat anti-mouse IgG reagents were applied to a strip of nitrocellulose membrane (Millipore, Boston, MA, USA) as a test line and control line at the target and control zones, respectively. After the membrane dried, it was cut into single test strips. The amounts applied to each strip were 1.5 μL of SSd–BSA and 1 μL of goat anti-mouse IgG. Each test strip was assembled by the collaboration of nitrocellulose membrane, an absorbent pad, the detection reagent in the conjugate pad, and a sample pad. 

A schematic diagram of the ICS assay is shown in Figure 1. The sample pads and conjugated pads, each made of the same type of glass fibers, were treated with suspend buffer contained 10 M Tris-HCl (pH = 8.8), 1% (*w*/*v*) BSA, 0.5% (*v*/*v*) Tween 20, and 1% (*v*/*v*) PEG 20000 for 1 min. The pads were air-dried in an incubator at 37 °C and kept in a hermetic bag at 4 °C before use. Colloidal gold-labeled anti-SSd MAbs were dispensed onto the conjugate pad with gold spraying equipment (Jieyi, Shanghai, China). The anti-SSd MAb–AuNP conjugate pads were then lyophilized. 

As the schematic diagram of the ICS strip in Figure 1 shows, the nitrocellulose membrane was firstly pasted onto a PVC plate (in the detection zone), the anti-SSd MAb–AuNP conjugate pad was pasted on the sheet with a 2 mm overlap with the bottom of the nitrocellulose membrane, and the absorbent pad (filter paper) was pasted on the sheet with a 2 mm overlap with the top of the membrane. Then, the simple pad was pasted onto the PVC plate with a 2 mm overlap with the conjugate pad. Then, the assembled plate was compacted using the JY-EQ02 batch laminating system and cut into 3.5-mm-wide strips using a paper cutter. The prepared strips were sealed with desiccant and stored at 4 °C.

Fifty microliters of the sample solution were applied onto the sample pad. After 10 min, the samples had migrated upwards, and the results of the test were read by inserting the test strip into a portable strip reader. From the colloidal gold on the test and control lines, the photometric value was estimated.

**Figure 4:**
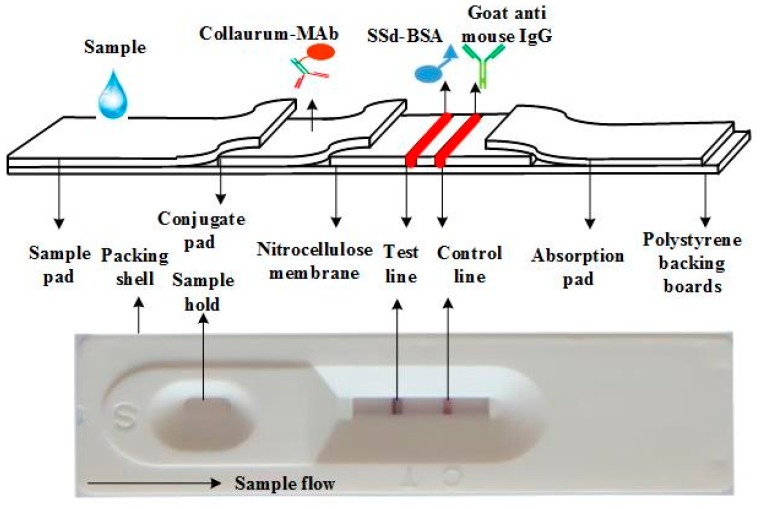
The structure and principle of the immunochromatographic strip (ICS). A nitrocellulose membrane, conjugate pad, sample pad, and absorbent pad were pasted onto a polyvinyl chloride backing to form the dipstick. Saikosaponin d (SSd)–bovine serum albumin (BSA) and goat anti-mouse IgG were used as the test capture reagent and control capture reagent, respectively. Using a dispenser, both the test and control capture reagents were dispensed separately as lines 0.5 cm apart on the nitrocellulose membrane.

The principle of the ICS assay is illustrated in Figure 1. The assay is based on a competitive immunoassay format. During the assay, a sample solution containing SSd was applied onto the sample-loading pad. The solution migrated towards the other end of the strip due to the capillary action driven by the absorbent paper (filter paper). As the sample solution reached the conjugation pad, SSd (antigen) reacted with the anti-SSd MAb–AuNPs pre-loaded on this pad. When the solution migrated along the strip and reached the test line, the unbound anti-SSd MAb–AuNP conjugates were selectively captured by the antigen (SSd–BSA) conjugate immobilized in this test zone. Then, the solution continued to migrate to the control line, where the SSd-antibody–AuNP conjugates and excess unbound anti-SSd MAb–AuNP conjugates were captured by the goat anti-mouse IgG conjugates immobilized in this zone. The test line will disappear when the quantity of SSd is sufficient compared to that of anti-SSd MAb–AuNPs. The entire process requires 5–10 min. As shown in Figure 4, upon visual inspection, it can be seen that the positive samples had only 1 red line (control line), but a negative test resulted in 2 red lines (test and control lines). The test was considered invalid if no control line was present. Next, the strips were analyzed by a portable strip reader (Shanghai Jieyi Biological Technology Co., Ltd.), which can provide the ratio of the test line to the control line (T/C).

#### 3.2.6. Specificity and Sensitivity of the ICS Test

We tested several different analogues of SSd, such as saikosaponin c (SSc), saikosaponin b2 (SSb2), and saikosaponin b1 (SSb1), to evaluate the specificity of the ICS test, with SSd used as a positive control and pure water used as a negative control. To evaluate the sensitivity, SSd standard solutions at concentrations of 150,000, 60,000, 12,000, 2400, 480 and 96 ng/mL were prepared by diluting SSd with distilled water, with each diluted sample applied to the ICS test. The visual limit of detection was defined as the minimum SSd concentration that resulted in the complete disappearance of the test line.

#### 3.2.7. Recovery Rate, Repeatability, and Stability of the ICS Assay

In the recovery experiment, various concentrations of SSd standard were added to distilled water or 100 ng/mL SSd. The contents of SSd in all the samples were assayed using ICS to determine the recovery rate.

The same batch of ICS assay strips was stored for 4 and 8 weeks and then used to detect SSd standard solutions containing several known concentrations of SSd to evaluate the stability of the assay. All strips were sealed with desiccant and stored at room temperature (25 °C).

#### 3.2.8. Analysis of the Consistency between the ICS Assay and the Indirect Competitive ELISA (icELISA)

To compare the ICS assay with icELISA, four ready-made herbal medicines including hu-gan-pian, xiao-chai-hu-ke-li, jia-wei-xiao-yao-wan, and xie-gan-wan were applied. These samples contain several herbs and various types of compounds, which can test the resistance of the ICS to interference.

The icELISA was performed as previously described [39]. Briefly, 100 μL of SSd–BSA were placed onto a microtiter plate, and incubation followed. Wells were blocked using 5% (*w*/*v*) skimmed milk. Then, sample or standard and an equal volume of anti-SSd–MAb ascitic fluid (1:8000) were added to the wells, and incubation followed. After another washing step, HRP-labeled goat anti-mouse IgG was added, and incubated for 30 min. Afterward, plates were washed, and 3,3′,5,5′-tetramethylbenzidine substrate solution was added to each well. After incubation, the reaction was stopped by a stop solution (21% sulfuric acid aqueous solution). Finally, the absorbance was measured at 450 nm (A450) on a spectrophotometric microplate reader.

## 4. Conclusions

In this study, a novel ICS using anti-SSd MAbs–AuNP conjugate was developed to rapidly detect SSd. TEM scanning showed that the AuNPs were spherical and uniform, with a diameter of approximately 18 nm. The ICS assay showed a high specificity for SSd. The sensitivity and accuracy of the ICS assay were also highly suitable for detecting SSd in practical use. To our knowledge, this is the first report of the quantitative detection of SSd by an ICS, which is a low-cost, on-site, and user-friendly method for detecting SSd in plant materials and biological samples. In conclusion, this method may provide an alternative tool in the analysis and quality control of SSd.

## Figures and Tables

**Table 1:** Cross-reactivity of compounds measured by ICS and indirect competitive ELISA (icELISA).

Samples	^a^ ICS (%)	^b^ icELISA (%)
SSd	100	100
SSa	4.30%	4.97%
SSb1	<0.09	<0.09
SSb2	<0.09	<0.09
SSc	<0.09	<0.09

The specificity of the ICS was tested by ICS and icELISA by evaluating reactivity with other compounds. ^a^ Cross-reactivity of samples measured by ICS; ^b^ Cross-reactivity of compounds measured by icELISA.

**Table 2:** Recovery rate of SSd.

SSd Concentration (ng/mL)	SSd Concentration Established by the Test System (ng/mL)	Recovery (%)
100	135.72 ± 61.97	135.72 ± 61.97
1000	926.59 ± 114.24	92.66 ± 11.42
10,000	11,128.16 ± 745.75	111.28 ± 14.12

Data are mean ± SD from triplicate samples at each spiked concentration of SSd. The percentage of recovery was calculated as follows: recovery (%) = measured amount/amount × 100%.

**Table 3:** Variations among ICS used for the analysis of SSd.

Sample	RSD %		
SSd (ng/mL)	1 Day ^a^	4 Weeks ^b^	8 Weeks ^c^
125	2.41	3.11	3.51
250	2.52	4.00	3.52
500	2.44	3.00	5.20
1000	3.12	2.71	4.50

^a^ The values indicate coefficients of variance for triplicate samples on three different strips used one day after manufacture; ^b^ The values indicate coefficients of variance for triplicate samples on three different strips used after being stored for four weeks; ^c^ The values indicate coefficients of variance for triplicate samples on three different strips used after being stored for eight weeks.

**Table 4:** Samples assayed by ICS and icELISA.

Sample	icELISA (mg/g)	ICS (mg/g)
hu-gan-pian	61.25 ± 11.25	51.74 ± 5.27
xiao-chai-hu-ke-li	735.25 ± 1.45	705.61 ± 1.13
jia-wei-xiao-yao-wan	182.315 ± 5.56	175.33 ± 1.27
long-dan-xie-gan-wan	138.55 ± 6.47	131.77 ± 8.89

Contents of total saikosaponins in four samples of Radix Bupleuri determined by icELISA with anti-SSd MAb and ICS. All data are presented as mean ± SD from triplicate wells analysed for each sample.

## Supplementary Materials

The supplementary materials are available online.

## Abbreviations

AuNPs	gold nanoparticles
LFIA	lateral flow immunoassay
MAb	monoclonal antibody
SSd	saikosaponin d
GICA	gold immunochromatographic assay
ICS	immunochromatographic strip
icELISA	indirect competitive ELISA
RB	Radix Bupleuri

## References

[1] Ackerson C.J., Powell R.D., Hainfeld J.F. (2010). Site-specific biomolecule labeling with gold clusters. Methods Enzymol..

[2] Xu C., Li X., Liu G., Xu C., Xia C., Wu L., Zhang H., Yang W. (2015). Development of ELISA and Colloidal Gold-PAb Conjugate-Based Immunochromatographic Assay for Detection of Abrin-a. Monoclon. Antib. Immunodiagn. Immunother..

[3] Paciotti G.F., Myer L., Weinreich D., Goia D., Pavel N., McLaughlin R.E., Tamarkin L. (2004). Colloidal gold: A novel nanoparticle vector for tumor directed drug delivery. Drug Deliv..

[4] Shi J., Votruba A.R., Farokhzad O.C., Langer R. (2010). Nanotechnology in drug delivery and tissue engineering: From discovery to applications. Nano Lett..

[5] Al-Dubai H., Lichtscheidl I., Strobl M., Pittner G., Pittner F. (2010). Immunosorbent assay using gold colloid cluster technology for determination of IgEs in patients’ sera. Nanotechnol. Sci. Appl..

[6] Ling S., Wang R., Gu X., Wen C., Chen L., Chen Z., Chen Q.A., Xiao S., Yang Y., Zhuang Z. (2015). Rapid detection of fumonisin B1 using a colloidal gold immunoassay strip test in corn samples. Toxicon.

[7] Wang L., Kong W., Yang M., Han J., Chen S. (2015). Safety issues and new rapid detection methods in traditional Chinese medicinal materials. Acta Pharm. Sin. B.

[8] He L., Nan T., Cui Y., Guo S., Zhang W., Zhang R., Tan G., Wang B., Cui L. (2014). Development of a colloidal gold-based lateral flow dipstick immunoassay for rapid qualitative and semi-quantitative analysis of artesunate and dihydroartemisinin. Malar. J..

[9] Wu J.X., Zhang S.E., Zhou X.P. (2010). Monoclonal antibody-based ELISA and colloidal gold-based immunochromatographic assay for streptomycin residue detection in milk and swine urine. J. Zhejiang Univ. Sci. B.

[10] Bruno J.G. (2014). Application of DNA Aptamers and Quantum Dots to Lateral Flow Test Strips for Detection of Foodborne Pathogens with Improved Sensitivity versus Colloidal Gold. Pathogens.

[11] Zhang L., Li D., Liu L., Zhang G. (2015). Rapid immunochromatographic test strip to detect swimming crab Portunus trituberculatus reovirus. Dis. Aquat. Organ..

[12] Kim Y.R., Park S.B., Fagutao F.F., Nho S.W., Jang H.B., Cha I.S., Thompson K.D., Adams A., Bayley A., Jung T.S. (2015). Development of an immunochromatography assay kit for rapid detection of ranavirus. J. Virol. Methods.

[13] Guo D.L., Pan Q.W., Li K.P., Li J.Q., Shen H.W., Wang X.L., Zhang X.Y., Li X.S., Fu F., Feng L. (2015). Development and clinical evaluation of a new gold-immunochromatographic assay for the detection of antibodies against field strains of pseudorabies virus. J. Virol. Methods.

[14] Zhang Y., Wang Y., Meng J., Xie Z., Wang R., Kutcher H.R., Guo Z. (2015). Development of an immunochromatographic strip test for rapid detection of lily symptomless virus. J. Virol. Methods.

[15] Zhang Y., Wang Y., Yang W., Xie Z., Wang R., Kutcher H.R., Guo Z. (2015). A rapid immunochromatographic test to detect the lily mottle virus. J. Virol. Methods.

[16] Zhang L., Li D., Liu L., Fang J., Xu R., Zhang G. (2015). Development of a colloidal gold immunochromatographic strip for the rapid detection of soft-shelled turtle systemic septicemia spherical virus. J. Virol. Methods.

[17] Liu X., Xiang J.J., Tang Y., Zhang X.L., Fu Q.Q., Zou J.H., Lin Y. (2012). Colloidal gold nanoparticle probe-based immunochromatographic assay for the rapid detection of chromium ions in water and serum samples. Anal. Chim. Acta.

[18] Wang Y., Wang L., Zhang J., Wang G., Chen W., Chen L., Zhang X. (2014). Preparation of colloidal gold immunochromatographic strip for detection of Paragonimiasis skrjabini. PLoS ONE.

[19] Putalun W., Tanaka H., Shoyama Y. (2005). Rapid detection of glycyrrhizin by immunochromatographic assay. Phytochem. Anal..

[20] Putalun W., Morinaga O., Tanaka H., Shoyama Y. (2004). Development of a one-step immunochromatographic strip test for the detection of sennosides A and B. Phytochem. Anal..

[21] Putalun W., Fukuda N., Tanaka H., Shoyama Y. (2004). A one-step immunochromatographic assay for detecting ginsenosides Rb1 and Rg1. Anal. Bioanal. Chem..

[22] Xiang T., Jiang Z., Zheng J., Lo C., Tsou H., Ren G., Zhang J., Huang A., Lai G. (2012). A novel double antibody sandwich-lateral flow immunoassay for the rapid and simple detection of hepatitis C virus. Int. J. Mol. Med..

[23] Huang X., Aguilar Z.P., Xu H., Lai W., Xiong Y. (2016). Membrane-based lateral flow immunochromatographic strip with nanoparticles as reporters for detection: A review. Biosens. Bioelectron..

[24] Lu C.N., Yuan Z.G., Zhang X.L., Yan R., Zhao Y.Q., Liao M., Chen J.X. (2012). Saikosaponin a and its epimer saikosaponin d exhibit anti-inflammatory activity by suppressing activation of NF-kappaB signaling pathway. Int. Immunopharmacol..

[25] Chao Z., Zeng W., Liao J., Liu L., Liang Z., Li X. (2014). DNA barcoding Chinese medicinal Bupleurum. Phytomedicine.

[26] Chiang L.C., Ng L.T., Liu L.T., Shieh D.E., Lin C.C. (2003). Cytotoxicity and anti-hepatitis B virus activities of saikosaponins from Bupleurum species. Planta Med..

[27] Law B.Y., Mo J.F., Wong V.K. (2014). Autophagic effects of Chaihu (dried roots of Bupleurum Chinense DC or Bupleurum scorzoneraefolium WILD). Chin. Med..

[28] Li X., Jin Y.Y., Zhang Y. (2009). Advances in mechanisms of saikosaponins in preventing and treating liver disease. Chin. J. Integr. Tradit. West. Med..

[29] Wong V.K., Zhou H., Cheung S.S., Li T., Liu L. (2009). Mechanistic study of saikosaponin-d (Ssd) on suppression of murine T lymphocyte activation. J. Cell. Biochem..

[30] Ying Z.L., Li X.J., Dang H., Wang F., Xu X.Y. (2014). Saikosaponin-d affects the differentiation, maturation and function of monocyte-derived dendritic cells. Exp. Ther. Med..

[31] Wong V.K., Li T., Law B.Y., Ma E.D., Yip N.C., Michelangeli F., Law C.K., Zhang M.M., Lam K.Y., Chan P.L. (2013). Saikosaponin-d, a novel SERCA inhibitor, induces autophagic cell death in apoptosis-defective cells. Cell Death Dis..

[32] Guan X., Wang X., Yan K., Chu Y., Li S., Li W., Yan X., Ma X., Zhou S., Sun H. (2016). UFLC-MS/MS determination and pharmacokinetic studies of six Saikosaponins in rat plasma after oral administration of Bupleurum Dropping Pills. J. Pharm. Biomed. Anal..

[33] Sai J., Zhao Y., Shan W., Qu B., Zhang Y., Cheng J., Qu H., Wang Q. (2016). Development of an Enzyme-Linked Immunosorbent Assay and Immunoaffinity Column Chromatography for Saikosaponin d Using an Anti-Saikosaponin d Monoclonal Antibody. Planta Med..

[34] Stobiecka M., Deeb J., Hepel M. (2010). Ligand exchange effects in gold nanoparticle assembly induced by oxidative stress biomarkers: Homocysteine and cysteine. Biophys. Chem..

[35] Stobiecka M., Coopersmith K., Hepel M. (2010). Resonance elastic light scattering (RELS) spectroscopy of fast non-Langmuirian ligand-exchange in glutathione-induced gold nanoparticle assembly. J. Colloid Interface Sci..

[36] Zhang Z., Lin M., Zhang S., Vardhanabhuti B. (2013). Detection of aflatoxin M1 in milk by dynamic light scattering coupled with superparamagnetic beads and gold nanoprobes. J. Agric. Food Chem..

[37] Biagini R.E., Sammons D.L., Smith J.P., MacKenzie B.A., Striley C.A., Snawder J.E., Robertson S.A., Quinn C.P. (2006). Rapid, sensitive, and specific lateral-flow immunochromatographic device to measure anti-anthrax protective antigen immunoglobulin g in serum and whole blood. Clin. Vaccine Immunol..

[38] Qian K., Liang Y.Z., Yin L.P., Shao H.X., Ye J.Q., Qin A.J. (2015). Development and evaluation of an immunochromatographic strip for rapid detection of capsid protein antigen p27 of avian leukosis virus. J. Virol. Methods.

[39] Zhang Y., Qu H., Zeng W., Zhao Y., Shan W., Wang X., Wang Q. (2015). Development of an enzyme-linked immunosorbent assay and immunoaffinity chromatography for glycyrrhizic acid using an anti-glycyrrhizic acid monoclonal antibody. J. Sep. Sci..

