# Supplementary material for: A Highly Sensitive Immunochromatographic Strip Test for Rapid and Quantitative Detection of Saikosaponin d
Source: Molecules. 2018 Feb 6;23(2):338. doi: 10.3390/molecules23020338 (PMC6017486; doi:10.3390/molecules23020338)
Supplement: Supplementary file 1 [file molecules-23-00338-s001.pdf]

Supporting information for

A Highly Sensitive Immunochromatographic Strip Test for Rapid and Quantitative Detection of Saikosaponin d

Table S1 Variations among ELISA runs for the analysis of SSd

	CV%	
SSd (ng mL^{-1})	ELISA[1]	ICS[2]
500	3.76	2.49
1000	4.29	2.64
2000	3.47	4.38

[1]For the variations, measured values indicate mean ± SD for samples run in triplicate on three different microtiter plates. [2]For the variations, six strips were tested for each concentration.
